# Ultrasound-guided perfusion index adjustment for hemostatic outcomes after femoral artery access in vascular interventions: a randomized controlled trial

**DOI:** 10.3389/fmed.2026.1735224

**Published:** 2026-02-24

**Authors:** Qiaoling Weng, Weihua He, Qiong Jiang, Weibo Zhong, Tingyu He

**Affiliations:** 1 Nanchang University Second Affiliated Hospital, Nanchang, Jiangxi Province, China; 2 Department of Anesthesiology, Union Hospital, Tongji Medical College, Huazhong University of Science and Technology, Wuhan, China; 3 Department of Anesthesiology, Ganzhou People’s Hospital, Ganzhou, Jiangxi Province, China

**Keywords:** femoral artery, hemostasis, interventions, perfusion index, ultrasound, vascular

## Abstract

**Objective:**

To evaluate the effect of adjusting the perfusion index (PI) under ultrasound guidance on hemostasis at the femoral artery puncture site in patients undergoing vascular interventional therapy.

**Methods:**

A total of 98 patients undergoing vascular interventions at Ganzhou People’s Hospital between July 2025 and September 2025 were randomized into two groups. The Trial group (*n* = 50) received ultrasound-guided initial compression to identify the optimal puncture site and establish a target PI from the great toe; a pressure bandage was subsequently applied to maintain the PI at or below this target. The Control group (*n* = 48) underwent conventional compression based on clinical experience and assessment of the dorsalis pedis artery pulse. The outcomes measured included hemostasis success upon bandage removal, Visual Analog Scale (VAS) pain scores assessed by a senior clinician, and PI values.

**Results:**

The effectiveness of compression hemostasis at the puncture site was significantly improved in the Group T after compression hemostasis with a pressure bandage compared to the Group C (16% VS 41.7%; difference, 25.7%; RR, 95% CI, 0.384, 0.187 to 0.787, *p* = 0.005).

**Conclusion:**

The adjustment of the PI value under ultrasound guidance significantly outperforms hemostasis when compared to empirical blind assessments following vascular interventional therapy.

**Clinical trial registration:**

ChiCTR.org.cn, identifier (ChiCTR2500105617).

## 1 Introduction

Percutaneous vascular intervention is a well-established minimally invasive endovascular technique that creates a surgical pathway through percutaneous arterial puncture (1), allowing for the guidance of catheters, balloons, stents, and other instruments to the target site for precise diagnosis or treatment (2, 3). This technique is characterized by minimal trauma, rapid recovery, and high repeatability. After decades of development, it has emerged as one of the core methods for managing cardiovascular (4, 5) and oncological diseases (6, 7). Due to its large diameter, fixed position, strong pulsation, and ease of puncture, the femoral artery is frequently utilized as the puncture site for vascular intervention procedures in clinical practice (8, 9). However, this approach has notable drawbacks. It can be challenging to achieve hemostasis and control bleeding upon catheter removal, which may lead to complications such as subcutaneous hematoma, hemorrhage, and even pseudoaneurysm (10). These complications can significantly hinder the patient’s postoperative recovery and may result in local ischemic necrosis of the lower limb, necessitating amputation (11). Therefore, effective hemostasis at the arterial puncture site postoperatively is a critical step in preventing hematoma and hemorrhage, thereby positively impacting the patient’s prognosis.

Current domestic and international studies indicate that vascular sutures and closure devices can be utilized for femoral artery hemostasis (12, 13). However, due to their high technical requirements and significant costs, these methods have not been widely adopted in clinical practice. Some studies even suggest that the use of hemostatic closure devices may lead to greater complications compared to manual compression hemostasis (14). Research by Sauer et al. demonstrates that pressure bandage hemostasis may be a safe and effective alternative (15), as it is simple to implement and cost-effective. Consequently, pressure bandage hemostasis remains commonly employed in clinical settings. Nonetheless, relying solely on the operator’s experience and the palpation of dorsalis pedis artery strength to regulate the tightness of the bandage for femoral artery hemostasis following vascular interventional therapy is insufficient for achieving effective hemostasis post-femoral artery puncture. Studies have shown (16) that excessive compression is detrimental to hemostasis. Prolonged high-pressure hemostasis may result in inadequate blood supply to the lower limbs, leading to ischemic necrosis. Therefore, there is an urgent clinical need for simpler and more effective judgment methods to provide patients with comfortable compression hemostasis techniques that ensure effective hemostatic outcomes.

Simultaneously, due to the anatomical positioning of the femoral artery, there exists a specific angle between the puncture needle and the skin during femoral artery puncture. Consequently, the bleeding point resulting from the femoral artery puncture is not perpendicular to the skin incision. Ultrasound imaging can be employed to visualize the bleeding point of the arterial puncture and to assess the success of hemostasis under ultrasound guidance. Therefore, ultrasound can be utilized to identify the site of hemostasis prior to attempting hemostatic measures. When tentative compression hemostasis is successful under ultrasound visualization, the PI value of the big toe can be determined (17) and utilized as a benchmark during the compression bandage hemostasis process. The compression force of the bandage can be calibrated to be less than or equal to this PI value, thereby allowing for the effective application of the PI value in post-femoral artery puncture hemostasis during compression bandage application.

Therefore, this study aims to utilize the PI value under ultrasound guidance to adjust the pressure of compression bandages and investigate the hemostatic effect in patients undergoing vascular interventional therapy guided by the PI value.

## 2 Methods

### 2.1 Study design

This study is a prospective randomized controlled trial approved by the hospital’s medical ethics committee (Ethics Approval Number: PJB2025-260-01) and has completed clinical trial registration (Registration Number: ChiCTR2500105617). Prior to the commencement of the trial, the trial protocol was thoroughly explained to the patients and their families, relevant treatment measures were discussed, and written informed consent was obtained from all participants. We adhered to the Consolidated Standards of Reporting Trials (CONSORT) reporting guidelines.

We recruited 118 adult patients who underwent vascular interventional therapy at Ganzhou People’s Hospital from July 2025 to September 2025. The inclusion criteria were as follows: ASA classification I–III; aged between 18 and 74 years; informed consent obtained from both patients and their families; and the right femoral artery designated as the catheterization site. The exclusion criteria included: poor compliance or inability to complete the study; patients deemed unsuitable for participation by the investigator; inability to monitor toe Pi values; presence of puncture-related lower extremity peripheral vascular disease; severe dysfunction of major organs, hematological or immune system diseases; abnormal coagulation function; and patients with a history of femoral artery puncture count ≥2. Additional exclusion criteria were patients who experienced massive bleeding necessitating the cessation of the procedure; and those whose surgical approach was altered during the operation. During the implementation phase, if a patient was uncooperative or voluntarily withdrew from the study, they would be considered as having dropped out.

#### 2.1.1 Randomization and blinding

**Figure 1:**
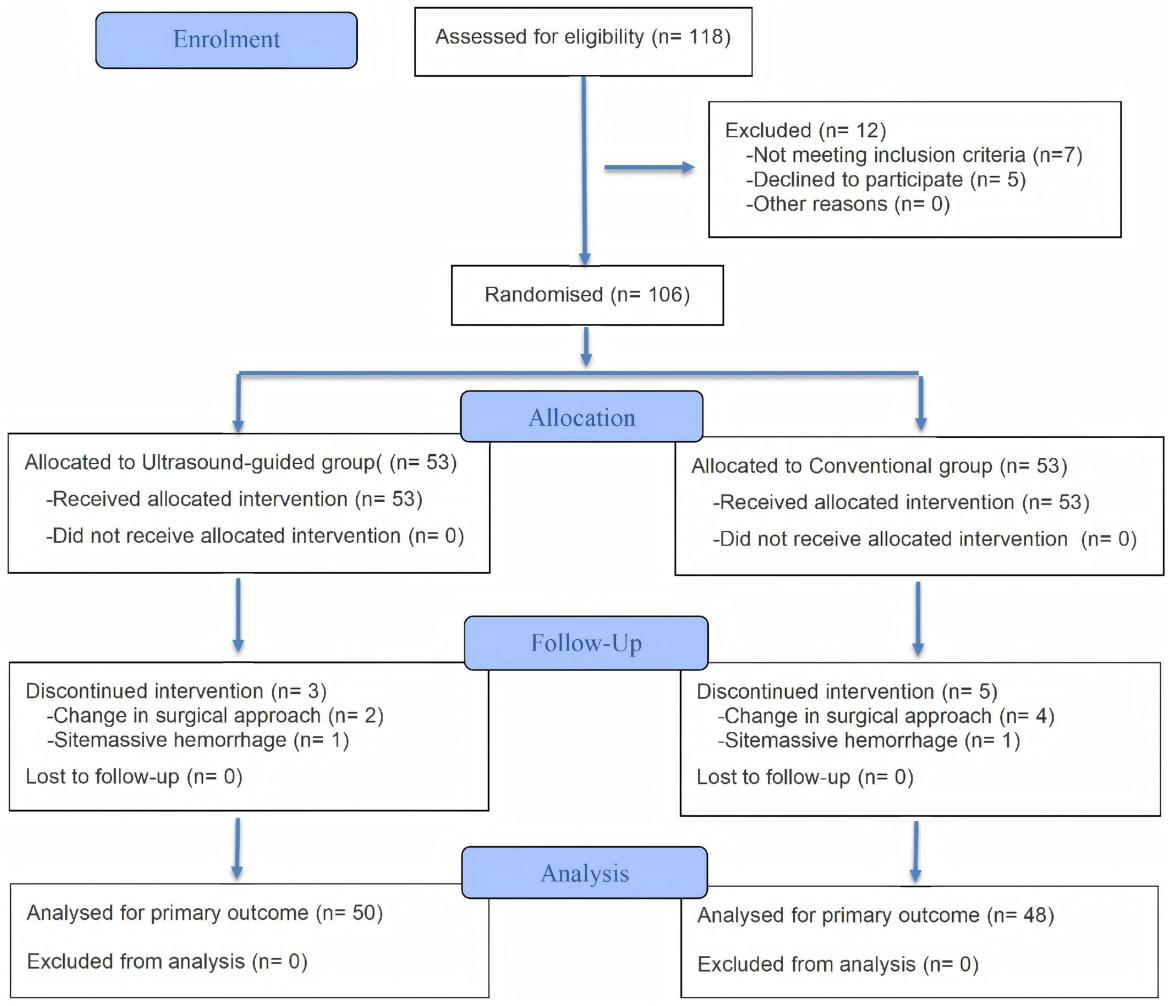
Flow diagram.

Using a computer-generated randomization table, participants were randomly assigned in a blinded manner in a 1:1 ratio to the ultrasound-guided group (Group T, *n* = 53) and the conventional group (Group C, *n* = 53), as illustrated in Figure 1. The study number and group allocation were kept in sealed envelopes by the same operating room nurse who was not involved in the study. Before performing compression hemostasis, the operating room nurse opened the sealed opaque envelope. Then, according to the grouping information in the envelope, the nurse informed the researcher to implement the corresponding intervention on the patients. The patients were unaware of the allocation of each group. The doctors (Qiong Jiang and Weibo Zhong) who participated in the postoperative follow-up blinded the grouping until the end of the experiment.

### 2.2 Preparations before hemostasis

All patients underwent vascular interventional therapy. Upon entering the room, they were connected to a monitor for the continuous monitoring of vital signs, including HR, SpO_2_, and non-invasive blood pressure, while peripheral venous access was established simultaneously. Continuous monitoring of PI value of the big toe was conducted for all patients. During measurement, it was ensured that the measurement site of the big toe cuff was properly fitted and immobilized. After the waveform stabilized, the PI value was recorded three times consecutively, and the average of these three measurements was calculated.

In this study, the right femoral artery puncture and catheterization for both the Group T and the Group C were performed by ultrasound-guided senior surgeons. If the number of puncture was more than 2, it was excluded from the study. All patients were punctured with 5F arterial puncture sheath. After successful puncture, heparin sodium injection was injected intravenously for anticoagulation treatment. The first dose was 60 IU/kg, and then 30 IU/kg was added every hour. Protamine antagonistic therapy was not used at the end of the operation.

Postoperatively, catheter removal and hemostasis for patients in both groups were managed by another experienced clinician. The hemostasis process was divided into two steps: initial compression hemostasis and a consolidation phase involving pressure bandage hemostasis. Patients in group t received ultrasound-guided femoral artery compression hemostasis, while patients in group C were operated by traditional touch method. All operations were performed by the same doctor to ensure the consistency of technical standards.

### 2.3 Hemostasis process

**Figure 2:**
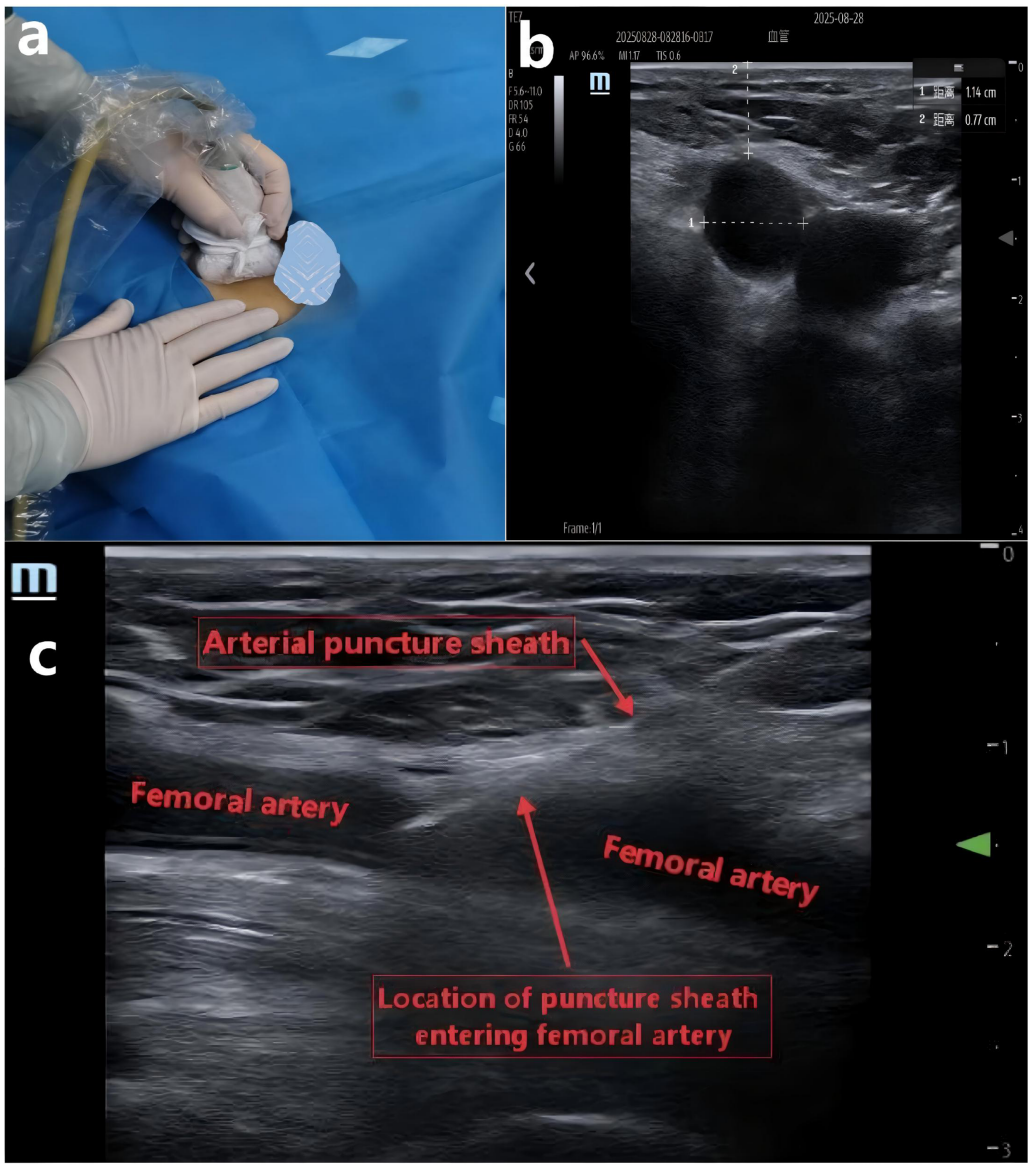
**(a)** Use ultrasound probe to compress and stop bleeding under ultrasound guidance. **(b)** Measure the diameter and depth of the femoral artery under ultrasound. **(c)** Ultrasound showed that the arterial puncture sheath entered the femoral artery.

In Group T, an ultrasound probe was utilized to guide compression hemostasis prior to the application of a pressure bandage postoperatively. Initially, ultrasound was employed to identify the bleeding site at the femoral artery puncture, marking it precisely above for accurate positioning during the subsequent application of the pressure bandage. The puncture catheter was then removed while maintaining compression with the ultrasound probe, which was used to compress the femoral artery bleeding site for hemostasis (as illustrated Figure 2). After 15 min, the hemostatic effect was evaluated. If hemostasis was not achieved, the procedure was repeated for an additional 15 min, and this process continued until successful hemostasis was accomplished (18), with the hemostasis time recorded. Throughout the hemostasis process, the PI_ultrasound_ value of the big toe was documented under direct ultrasound visualization when the bleeding site was accurately compressed and hemostasis was successful. This PI_ultrasound_ value was subsequently used as the maximum allowable PI value during the pressure bandage hemostasis. Following successful hemostasis under ultrasound guidance, a pressure bandage was applied directly above the marked puncture bleeding site to maintain hemostasis for 6–8 h, ensuring that the PI value remained ≤ the PI_ultrasound_ value, and the PI value at this time was recorded.

In Group C, hemostasis was achieved by applying pressure with hemostatic gauze, guided by clinical experience, and the PI value was subsequently recorded. After 15 min, the hemostatic effect was assessed. If hemostasis was not attained, the procedure was repeated for an additional 15 min, continuing this process until successful hemostasis was achieved (19). The duration of hemostasis was recorded, and the puncture site was monitored for any signs of bleeding. Once hemostasis was confirmed, the tightness of the femoral artery hemostatic compression bandage was adjusted according to the strength of the dorsal artery pulse, with compression maintained and Postoperative immobilization for 6–8 h. The PI value following bandaging was then recorded.

This study evaluates the VAS scores and the Kolcaba Comfort Scale scores (20), along with satisfaction rates and the incidence of pseudoaneurysm at the puncture site. Additionally, it examines occurrences of femoral artery embolism, right lower limb numbness, and bleeding at the puncture site following the removal of the compression bandage in both patient groups after initial compression hemostasis and pressure bandage hemostasis. Furthermore, operator satisfaction is recorded, and the hemostasis within 48 h after the operation at the puncture point was graded, which includes visible bleeding at the skin puncture site (bleeding at the puncture point again, which needs to extend the compression time), ecchymosis (diameter > 5 mm), and subcutaneous hematoma (indicating deep bleeding, diameter > 10 mm with or without skin elevation). The total incidence of the three complications is the compression hemostasis effect of the patient. All patients’ comfort score, pain score and satisfaction were followed up by the blind researcher (Qingming Zeng) to ensure the consistency of the evaluation criteria. The whole process is recorded by independent observers to ensure the accuracy of data collection and the standardization of operation.

### 2.4 Observation indexes

#### 2.4.1 General information

This study provides a comprehensive overview of the general conditions of patients in both groups, including age, gender, height, weight, SpO_2_, MAP, HR, ASA classification, platelet count, albumin level, hemoglobin level, white blood cell count, Pittsburgh Sleep Quality Index (PSQI) score, and Quality of Recovery (QoR-15) score. Additionally, it details surgical parameters such as operation time, blood loss, fluid infusion volume, and urine output, ensuring a robust analysis of the clinical context.

#### 2.4.2 Primary observation indicators

The effectiveness of compression hemostasis in both groups of patients was the total incidence of bleeding related complications within 48 h (incidence of bleeding, hematoma, and ecchymosis).

#### 2.4.3 Secondary observation indicators

1. The first hemostasis time, PI value detection rate, VAS score, Kolcaba comfort scale score, patient and operator satisfaction were measured in two groups of patients who were treated with ultrasound probe and hemostatic gauze compression hemostasis, respectively, (T1);
2. The detection rate of PI value, VAS score, Kolcaba comfort scale score, and satisfaction of patients and operators during pressure bandage hemostasis (T2) in the two groups of patients;
3. Complications that occurred within 30 days (T5) after surgery (incidence of pseudoaneurysm, femoral artery thrombosis, and lower limb numbness on the operated side);
4. PSQI scores were assessed at 30 days post-surgery (T5);
5. QoR-15 scores were assessed at 24 h (T3) and 48 h (T4) after surgery.

### 2.5 Sample size and statistical analysis

According to the preliminary experiment, the incidence of hematoma following vascular interventional therapy was observed to be 5% in Group T and 25% in Group C. With a two-sided *α* of 0.05 and a power of 1−β equal to 0.8, the calculated minimum sample size for the study was determined to be at least 94 patients. Considering potential changes in clinical conditions that might necessitate the exclusion of some patients, a total of 118 patients were initially recruited for the study. Categorical data are presented as counts or percentages, while quantitative data displaying a normal distribution are expressed as means and standard deviations. Parametric tests were employed for data with a normal distribution, whereas non-parametric tests were utilized for data that did not meet this criterion. The Chi-square test and Fisher’s exact probability test were applied to analyze categorical data. A *p*-value of less than 0.05 was established as the threshold for statistical significance. All data analyses were conducted using the Statistical Product and Service Solutions 26.0 (SPSS 26.0).

## 3 Results

Among the 118 eligible patients selected, 12 patients were excluded according to the exclusion criteria. A total of 106 patients who met the inclusion criteria were randomly assigned to two groups using a computer-generated randomization table: the group guided by the PI value for femoral artery hemostasis after vascular interventional therapy with ultrasound assistance (Group T, *n* = 53) and the group guided by the assessment of dorsalis pedis artery pulsation for femoral artery hemostasis after vascular interventional therapy (Group C, *n* = 53). Due to changes in surgical methods and major intraoperative bleeding, ultimately, 50 patients in Group T and 48 patients in Group C ultimately met the criteria (as illustrated Figure 1).

### 3.1 Comparison of general conditions between the two groups of patients

There were no statistically significant differences in general conditions such as age, gender, height, weight, oxygen saturation, sleep index, recovery index, heart rate, mean arterial pressure, platelet count, and hemoglobin between Group T and Group C patients (*p* > 0.05) (Table 1).

**Table 1:** Demographic and perioperative characteristics.

Characteristic	Patients, mean (IQR)		Z/T/χ^2^	p
	Group T (*n* = 50)	Group C (*n* = 48)		
Age, [range], y	57.86 (8.57) [40–71]	55.56 (9.12) [34–74]	−1.355	0.175
Height, (SD), cm	161.20 (8.38)	161.90 (8.17)	−0.995	0.322
Weight, kg	60 (55–65.25)	60.5 (54.25–69.5)	−0.961	0.337
BMI, (SD), kg/m^2^	23.17 (3.47)	23.88 (3.59)	−0.416	0.678
Diameter, mm	9.05 (7.95–9.80)	9.25 (7.60–9.87)	−0.484	0.629
Depth, mm	11.35 (9.20–14.60)	13.25 (10.80–15.20)	−1.546	0.122
PI value	2.92 (2.58–3.27)	2.81 (2.60–3.33)	−0.505	0.614
WBC, 10^9^/L	6.95 (5.11–8.10)	6.10 (5.12–6.95)	−1.755	0.079
HGB, g/L	129 (120–143.25)	134.5 (124–143)	−0.1.049	0.294
PLT, 10^9^/L	243.5 (189.25–276.5)	217.5 (195–247.75)	−1.706	0.088
SpO_2_, %	97 (97–98)	97 (96–98)	−1.601	0.109
Dosage of heparin sodium injection, IU	3,600 (3300–3,915)	3,630 (3255–4,170)	−0.961	0.337
Operative time, min	49 (42–55)	51 (45–57.75)	−1.174	0.241
Puncture times	1 (1–1)	1 (1–1)	−0.284	0.776
Diabetes, No. (%)	12 (24.0)	17 (35.4)	1.532	0.216
Hypertension, No. (%)	32 (64.0)	30 (62.5)	0.024	0.878
Gender				
Male, No. (%)	34 (68.0)	34 (70.8)	0.093	0.761
Female, No. (%)	16 (32.0)	14 (29.2)		

Group T: the group guided by the PI value for femoral artery hemostasis, group C: the group guided by the assessment of dorsalis pedis artery pulsation. SD, Standard deviation; IQR, Interquartile range; BMI, Body Mass Index; PLT, Platelet; HGB, Hemoglobin; WBC: White blood cells; SpO_2_: Pulse Oxygen Saturation. Compared with group C, ^*^p < 0.05, ^**^p < 0.05.

### 3.2 Comparison of the primary endpoint between the two groups

**Figure 3:**
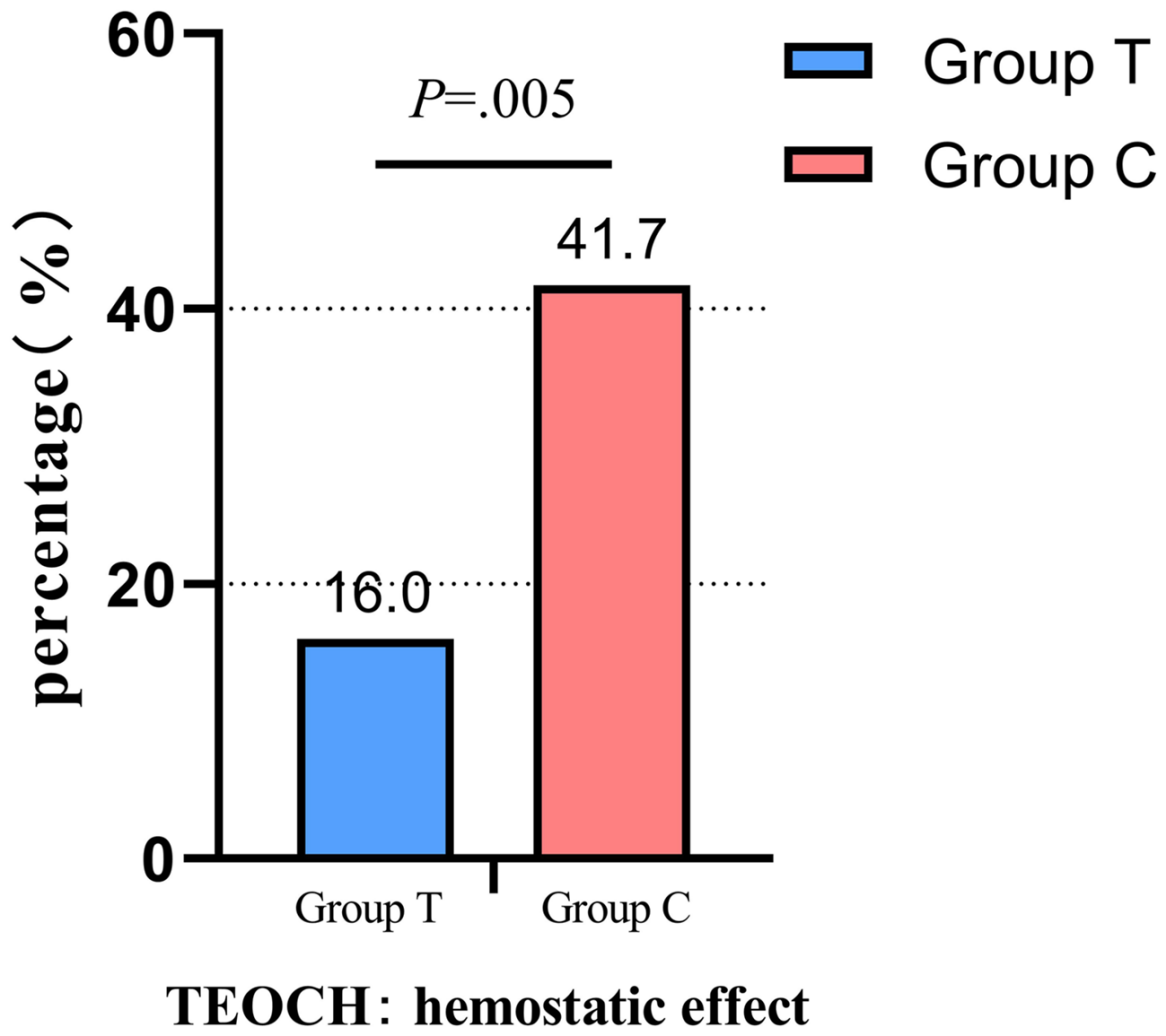
Group T: the group guided by the PI value for femoral artery hemostasis, group C: the group guided by the assessment of dorsalis pedis artery pulsation. TEOCH, The effectiveness of compression hemostasis: the total incidence of three indicators related to hemostatic effect; Comparison of hemostatic effects between the two patient groups.

The findings of the study indicate that compared with group C, the hemostatic effect of group T was significantly improved (16% VS 41.7%; difference, 25.7%; RR, 95% CI, 0.384, 0.187 to 0.787, *p* = 0.005) (Table 2; Figure 3).

**Table 2:** Comparison of primary endpoint, initial hemostasis time, adverse reactions and satisfaction.

Variable	Patients, No. (%)		*χ* ^2^	p	RR (95% CI)
	Group T (*n* = 50)	Group C (*n* = 48)			
Primary endpoint					
TEOCH	8 (16)^*^	20 (41.7)	7.905	0.005	0.384 (0.187–0.787)
Secondary endpoint					
Bleeding	0 (0)	1 (2.1)	1.052	0.305	NA
Ecchymosis	6 (12)	11 (22.9)	2.036	0.154	0.524 (0.210–1.304)
Hematoma	2 (4)^*^	8 (16.7)	4.288	0.038	0.240 (0.054–1.073)
Pseudoaneurysm	0 (0)	0 (0)	NA	>0.999	NA
Femoral artery embolization	0 (0)	0 (0)	NA	>0.999	NA
Numbness of lower limbs	1 (2.0)	2 (4.2)	0.387	0.534	0.480 (0.045–5.122)
Patient satisfaction	39 (78.0)^*^	27 (56.3)	5.268	0.022	1.387 (1.038–1.853)
Satisfaction of doctors	44 (88.0)^*^	34 (70.8)	4.443	0.035	1.242 (1.009–1.530)
Hemostasis time, median (IQR)	15 (15–15)^*^	15 (15–30)	4.186	0.041	NA

Group T: the group guided by the PI value for femoral artery hemostasis, group C: the group guided by the assessment of dorsalis pedis artery pulsation. TEOCH, The effectiveness of compression hemostasis: the total incidence of three indicators related to hemostatic effect; cIQR, Interquartile range; RR, relative risk; CI, Confidence Interval. Compared with group C, ^*^p < 0.05, ^**^p < 0.05.

### 3.3 Comparison of PI value detection rate, VAS score and Kolcaba comfort scale score between the two groups of patients

**Figure 4:**
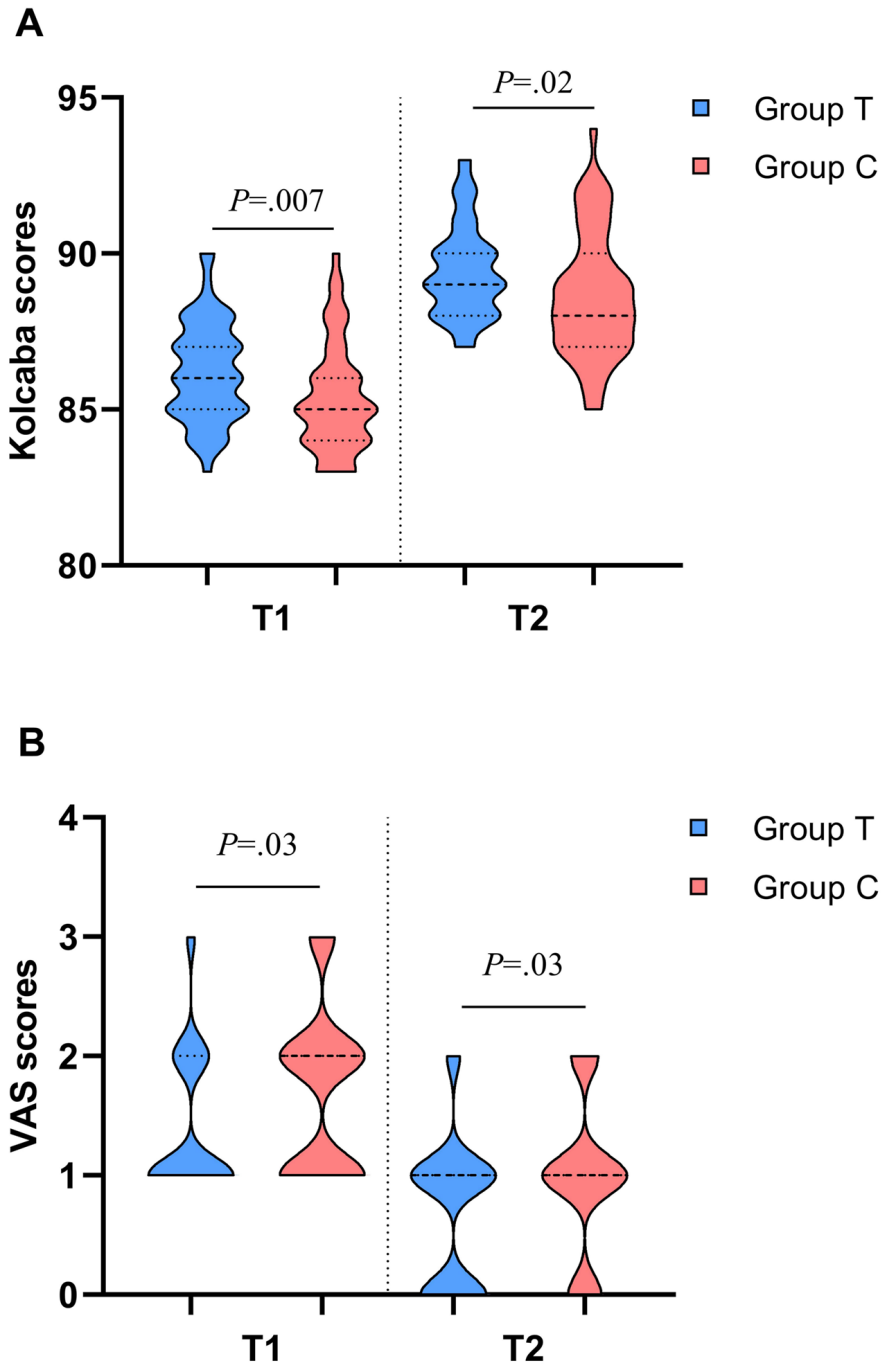
Group T: the group guided by the PI value for femoral artery hemostasis; Group C: the group guided by the assessment of dorsalis pedis artery pulsation. VAS, Visual Analogue Scale. T1, first hemostasis; T2, when applying a pressure bandage to stop bleeding. **(a)** Comparison of Kolcaba scores between the two groups of patients. **(b)** Comparison of VAS scores between the two groups of patients.

The detection rate of PI in Group T was significantly higher than that in Group C during the first hemostasis (*p* < 0.001), while the detection rates of PI during compression bandage hemostasis were 86 and 66.7%, respectively, (*p* = 0.024). The Kolcaba scores of patients in Group T were higher than those in Group C during both the first hemostasis and compression bandage hemostasis, and the VAS comfort scale scores were significantly lower than those in Group C, with statistically significant differences (*p* < 0.05) (Table 3; Figure 4).

**Table 3:** Comparison of PI value detection rate, VAS score and Kolcaba comfort scale score.

Variable	Patients, median (IQR)		Z/χ^2^	p
	Group T (*n* = 50)	Group C (*n* = 48)		
Comparison of PI value detection rates between two groups, No. (%)				
T1	50 (100.0)^**^	23 (47.9)	34.960	<0.001
T2	43 (86.0)^*^	32 (66.7)	5.096	0.024
Comparison of VAS scores between two groups at different time points				
T1	1 (1–2)^*^	2 (1–2)	−2.224	0.026
T2	1 (1–1)^*^	1 (1–2)	0.2.115	0.034
Comparison of Kolcaba scores between two groups at different time points				
T1	86 (85–87)^*^	85 (84–86)	−2.678	0.007
T2	89 (88–90)^*^	88 (87–90)	−2.337	0.019

Group T: the group guided by the PI value for femoral artery hemostasis, group C: the group guided by the assessment of dorsalis pedis artery pulsation. IQR, Interquartile range; VAS, Visual Analogue Scale; PI, Perfusion index. Compared with group C, ^*^p < 0.05, ^**^p < 0.05.

### 3.4 Comparison of initial hemostasis time, adverse reactions and satisfaction between the two groups of patients

The time to initial hemostasis in Group T was significantly shorter than that in Group C (*p* = 0.041); there were no statistically significant differences in the incidence of pseudoaneurysms, femoral artery embolism, and numbness in the lower limb on the operated side after femoral artery hemostasis (*p* > 0.05); however, patient satisfaction and doctor satisfaction in Group T were significantly higher than those in Group C (*p* < 0.05) (Table 2).

### 3.5 Comparison of hemodynamic parameters between the two groups of patients

There was no statistically significant difference in mean arterial pressure and pulse rate between the two groups at different time points (*p* > 0.05) (Table 4).

**Table 4:** Comparison of hemodynamic parameters between the two groups of patients.

Variable	T0	T1	T2	T3	T4
MAP of patients at various time points, mean (SD), mmHg					
T (*n* = 50)	94.96 (11.07)	99.18 (11.42)	96.56 (11.31)	93.28 (11.27)	91.36 (11.06)
C (*n* = 48)	96.94 (11.83)	103.00 (12.18)	99.31 (12.31)	94.88 (11.84)	92.75 (11.42)
F	0.731	2.566	1.330	0.467	0.375
P	0.395	0.112	0.252	0.496	0.542
*P-*group	*F = 0*.987	*p* = 0.323			
*P-*time	*F =* 508.984	*P* < 0.001			
*P-*group × time	*F =* 10.311	*P* < 0.001			
HR of patients at various time points, mean (SD), bpm					
T (*n* = 50)	72.72 (10.02)	79.78 (10.28)	76.84 (10.31)	72.90 (8.98)	70.12 (9.97)
C (*n* = 48)	72.54 (8.21)	82.00 (7.76)	78.23 (7.55)	73.58 (8.02)	70.44 (7.65)
F	0.009	1.447	0.575	0.158	0.031
P	0.924	0.232	0.450	0.692	0.860
*P-*group	*F = 0*.251	*p* = 0.618			
*P-*time	*F =* 393.716	*P* < 0.001			
*P-*group×time	*F =* 4.820	*p* = 0.008			

Group T: the group guided by the PI value for femoral artery hemostasis, group C: the group guided by the assessment of dorsalis pedis artery pulsation. SD, Standard deviation; MAP, Mean arterial pressure; HR, heart rate. Compared with group C, ^*^p < 0.05, ^**^p < 0.05.

### 3.6 Comparison of sleep quality index (PSQI) and recovery index (QoR-15) between the two groups of patients

There was no statistically significant difference in PSQI scores between the two groups at 30 days post-surgery (*p* > 0.05), and there were also no significant differences in QoR-15 scores at 24 h and 48 h post-surgery (*p* > 0.05) (Table 5).

**Table 5:** Comparison of sleep quality index (PSQI) and recovery index (QoR-15) between the two groups of patients.

Variable	Patients, median (IQR)		Z	p
	Group T (*n* = 50)	Group C (*n* = 48)		
Comparison of QoR-15 scores between two groups at different time points				
T0	142 (141–145)	143 (141–145)	−0.561	0.575
T3	127 (120–133)	125 (119–130)	−1.455	0.146
T4	132 (129–138)	133 (129–139)	−0.510	0.610
T5	144 (143–147)	144 (143–147)	−0.316	0.752
Comparison of PSQI scores between two groups at different time points				
T0	7 (5–8)	6 (5–8)	−0.245	0.807
T5	6 (5–7)	6 (4–7)	−0.340	0.734

Group T: the group guided by the PI value for femoral artery hemostasis, group C: the group guided by the assessment of dorsalis pedis artery pulsation. IQR, Interquartile range; QoR-15, Quality of Requirements-15; PSQI, Pittsburgh Sleep Quality Index. Compared with group C, ^*^p < 0.05, ^**^p < 0.05.

### 3.7 Comparison of bleeding related complications between two groups of patients

There was no significant difference in the incidence of bleeding and ecchymosis between Group T and Group C patients (*p* > 0.05), while the incidence of hematoma was 4 and 16.7%, respectively, with a statistically significant difference (*p* = 0.038) (Table 2).

## 4 Discussion

This study aims to investigate the effectiveness of adjusting the PI value under ultrasound guidance in achieving hemostasis after vascular interventional therapy. The goal is to guide clinicians in performing more accurate, effective, and complication-free femoral artery hemostasis, thereby enhancing patient satisfaction in individuals undergoing post-vascular interventional therapy. The findings of the study indicate that compared with group C, the hemostatic effect of group T was significantly improved (16% VS 41.7%; difference, 25.7%; RR, 95% CI, 0.384, 0.187 to 0.787, *p* = 0.005). And during the initial compression hemostasis process, the Group T experienced a significantly shorter compression hemostasis time (*p* = 0.041), along with increased satisfaction levels among both patients and hemostasis operators (*p* < 0.05).

During the evaluation of hemostasis with pressure bandages in patients undergoing vascular interventional therapy, it was observed that Group T achieved a 100% detection rate of the PI value in the big toe of the right limb during the initial pressure hemostasis (*p* < 0.001), compared to Group C. Additionally, Group T demonstrated a higher detection rate during subsequent pressure bandage hemostasis (*p* = 0.021), further indicating that ultrasound-guided hemostasis exerts a lesser impact on lower limb circulation. Although current research suggests that the preferred site for monitoring the PI value is the finger (21), this study emphasizes the importance of evaluating the effectiveness and complications associated with femoral artery hemostasis. Furthermore, under ultrasound guidance, the puncture site can be precisely selected (22), resulting in a higher success rate for the first puncture and fewer complications compared to guidance based on anatomical landmarks (23). This method also facilitates more accurate localization of the bleeding point at the femoral artery puncture site (24). In the initial hemostasis assessment prior to the application of pressure bandages in both patient groups, it was confirmed that the ultrasound-guided hemostasis method resulted in a shorter hemostasis time than the blind manual compression method (*p* = 0.041), which may play a crucial role in the management of pseudoaneurysms (25). Although this study revealed no statistically significant difference (*p* > 0.05) in the incidence of complications such as pseudoaneurysm, femoral artery embolism, and numbness in the operated lower limb between the two patient groups who received postoperative compression bandage hemostasis, and no significant difference was noted in the assessment of sleep and recovery indices (*p* > 0.05), the incidence of these complications was significantly lower in the group where the PI value was adjusted under ultrasound guidance. This finding suggests that excessive compression or inaccurate positioning during the manual compression process in the blind manual compression group may have contributed to the formation of pseudoaneurysm, femoral artery embolism, and numbness in the operated lower limb. It may lead to longer hemostasis time, later ambulation time, and even increase the incidence of deep venous thrombosis (DVT) (26). In contrast, ultrasound guidance may have mitigated these complications by ensuring adequate blood supply to the patient’s lower limb and fulfilling the requirements for visual localization of femoral artery hemostasis. Furthermore, it effectively reduced the pain VAS score during the initial compression hemostasis and subsequent compression bandage hemostasis (*p* < 0.05), potentially alleviating discomfort during the immobilization process of compression bandage hemostasis (27). This improvement could enhance patient compliance and satisfaction, decrease the likelihood of compression bandage displacement, and further optimize the postoperative femoral artery hemostasis effect in patients undergoing vascular interventional therapy, while also improving the satisfaction of the hemostasis operator. The adjustment of the PI value under ultrasound guidance has been validated to result in lower incidences of bleeding, hematoma, and ecchymosis in patients undergoing vascular interventional therapy who utilize pressure bandages for postoperative hemostasis. This finding confirms that adjusting the PI value under ultrasound guidance can facilitate more efficient hemostasis in these patients. Similarly, related studies have also investigated the hemostatic effects achieved through ultrasound guidance (28, 29). Although this study excludes patients who cannot monitor toe PI, which makes this study not applicable to some people, such as pad/clti patients, for patients who lack or are unreliable toe PI, ultrasound-guided hemostasis may still be a simple and effective option at present.

Although vascular closure devices (VCDs) have been widely used in many centers around the world (30–32). However, relevant studies still recommend the use of real-time ultrasound for vascular hemostasis and occlusion of VCDs, so as to reduce equipment failure and complications related to puncture site (33). At the same time, the starting point of choosing manual pressing as the research object is also based on the profound consideration of the real-world medical situation in China and many parts of the world: 1. In many medical institutions in low - and middle-income regions, the high cost restricts the conventional application of VCDs. Manual compression is still the most important hemostatic method in most hospitals, especially in primary and intermediate medical centers. 2. Not all patients are suitable or necessary to use VCDs. For example, the use of VCDs may increase the risk of complications in patients with abnormal anatomy of puncture site, severe vascular calcification, or peripheral arterial disease. Manual compression is a universal technology, which is applicable to almost all patients. To study how to improve this “bottom covering” technology is an important reinforcement of the patient safety net. The original intention of this study is not to deny or replace VCDs, but to solve the problem that in the vast scenes where manual pressing is still necessary, it is an important and practical supplement to the current existing technology. This article integrates the cost-effective and straightforward functionality of the pressure bandage hemostasis method with the widely adopted and standardized ultrasound visualization technology in clinical practice (34, 35), rendering the pressure bandage hemostasis method no longer blind and inefficient. For patients and clinicians facing the high costs and technical demands associated with new hemostatic technologies (36), adjusting the PI value under ultrasound guidance for hemostasis may present a valuable alternative method worth considering.

### 4.1 Limitations

The study has certain limitations. Firstly, our inclusion criteria excluded patients with coagulation disorders. Due to the limited research on this population, the applicability of the study’s conclusions to patients with coagulation disorders necessitates further exploration. Secondly, the experiment only compared the hemostasis method of adjusting the PI value under ultrasound guidance with the conventional clinical method of assessing the strength of the dorsalis pedis artery pulse by palpation. It did not include comparisons with newer hemostatic technologies, such as VCDs. Therefore, this trial cannot conclusively demonstrate that the hemostasis method of adjusting the PI value under ultrasound guidance is superior to newer technologies like vascular suturing devices. Further research is required for validation. Additionally, since the PI value may not yield results when a pressure bandage is applied, we used the detection rate to reflect changes in lower limb circulation. And there was variability in the patients’ pain VAS scores, as different patients may have varying pain thresholds during pressure hemostasis, which could influence the pain scores. Lastly, this study lacks the evaluation of patients with lower extremity deep venous thrombosis, and the evaluation of the complications of the two compression hemostasis methods may not be comprehensive enough.

## 5 Conclusion

Adjusting the PI value under ultrasound guidance effectively enhances hemostasis following vascular interventional therapy, reduces complications, and accelerates patient recovery.

## Data Availability

The original contributions presented in the study are included in the article/supplementary material, further inquiries can be directed to the corresponding authors.
